# Emotional impact of screening: a systematic review and meta-analysis

**DOI:** 10.1186/1471-2458-11-603

**Published:** 2011-07-28

**Authors:** Ruth E Collins, Laureen M Lopez, Theresa M Marteau

**Affiliations:** 1 Department of Psychology (at Guy's), Kings College London, Health Psychology Section, 5th Floor Bermondsey Wing, Guy's Campus, London, SE1 9RT, UK; 2 Clinical Sciences FHI, PO Box 13950, Research Triangle Park, NC 27709, USA

## Abstract

**Background:**

There is a widely held expectation that screening for disease has adverse emotional impacts. The aim of the current review is to estimate the short (< 4 weeks) and longer term (> 4 weeks) emotional impact of such screening.

**Methods:**

Studies selected for inclusion were (a) randomised controlled trials in which (b) participants in one arm underwent screening and received test results, and those in a control arm did not, and (c) emotional outcomes were assessed in both arms. MEDLINE via PubMed (1950 to present), EMBASE (1980 to present), PsycINFO (1985 to present) using OVID SP, and CINAHL (1982 to present) via EBSCO were searched, using strategies developed with keywords and medical subject headings. Data were extracted on emotional outcomes, type of screening test and test results.

**Results:**

Of the 12 studies that met the inclusion criteria, six involved screening for cancer, two for diabetes, and one each for abdominal aortic aneurysms, peptic ulcer, coronary heart disease and osteoporosis. Five studies reported data on anxiety, four on depression, two on general distress and eight on quality of life assessed between one week and 13 years after screening (median = 1.3 years).

Meta-analyses revealed no significant impact of screening on longer term anxiety (pooled SMD 0.01, 95% CI -0.10, 0.11), depression (pooled SMD -0.04, 95% CI -.12, 0.20), or quality of life subscales (mental and self-assessed health pooled SMDs, respectively: 0.03; -0.01, (95% CI -.02, 0.04; 0.00, 95% CI -.04, 0.03).

**Conclusion:**

Screening does not appear to have adverse emotional impacts in the longer term (> 4 weeks). Too few studies assessed outcomes before four weeks to comment on the shorter term emotional impact of screening.

## Background

Screening for disease and estimating disease risk using biomarkers such as cholesterol or blood pressure have formed a routine part of healthcare for over 50 years [1]. The term mass screening is often used when screening involves the examination of large populations or cohorts. The emotional impact of such screening was largely unquestioned until the publication of a paper presenting evidence of increased absenteeism following the detection of hypertension [2]. Since then there have been numerous reports of the emotional impact of screening upon anxiety, depression, intrusive and troubling thoughts, and, amongst children, absence from school and behavioural disturbance [3,4].

Screening is a complex event involving those targeted in a range of possible outcomes: awareness of screening; receipt of an invitation; undergoing a screening test; receiving a test result and concern regarding the test result (anxiety over a possible diagnosis as well as potential concern over false positive or false negative results). Awareness of screening appears not to induce emotional distress [5]. Receipt of an invitation for screening may, however, precipitate emotional distress [6] although this is not always the case [7]. More uncertainty and concern exist about the emotional impact of the latter two stages. Individuals can react with concern, anxiety and even depression when informed that they have an elevated risk of developing a disease [8]. While some emotional distress may be helpful, too much can be debilitating [9]. Not only can distress reduce individuals' quality of life, it can also interfere with information processing [10] and hence the ability to be reassured or to make informed choices regarding future treatment options. Emotional distress can also result in people avoiding future surveillance [11-13].

Existing reviews of the emotional impact of screening report few adverse effects on generalised and specific anxiety, depression or quality of life [3,14-17]. Whilst some studies report an increase in immediate emotional distress, few report distress that is sustained in the longer term. Furthermore, between-group analyses reveal few enduring effects of being identified as having an elevated risk of disease. For example, a meta- analysis assessing standardised differences between those receiving positive compared with negative test results revealed increased anxiety and depression within the first four weeks of receiving test results but no difference after four weeks [3]. These findings stand in contrast to a widespread belief amongst healthcare providers that screening for risk of disease has adverse emotional consequences [18,19].

The evidence from these reviews is, however, limited in two ways. First, only a minority included randomised controlled trials (RCTs) [3,15] and none restricted inclusion to RCTs, thereby limiting the ability to infer a causal connection between screening and emotional outcomes. Second, the dominant comparisons reported are non-randomised comparisons within screened groups, *i.e*. between those receiving test results indicating an elevated risk of disease (screen positive) and those receiving test results indicating no elevated risk (screen negative). While such comparisons are important for being able to assess and, where necessary, attempt to prevent excess distress, they do not address the question of interest from a population health perspective, namely, whether screening causes distress in populations invited and participating in screening programmes. Estimating this with precision requires comparisons between those randomised to undergo screening and those randomised not to undergo screening. We report here the first such meta-analytic comparison using data from randomised controlled trials.

The primary aim of this review is to estimate the immediate and longer term emotional impact of undergoing screening or risk assessment for disease. To improve upon existing reviews, we include only randomised controlled trials in which a principal analysis reports emotional outcomes of the randomised groups, *i.e*. trials where it is possible to compare emotional outcomes in those randomised to undergo screening or risk assessment with those randomised not to undergo such testing. The secondary aim is to estimate the emotional impact of receiving an elevated risk (screen positive) test result, by comparing outcomes of this subgroup with those receiving a non-elevated or average risk test result.

## Methods

### Data sources and searches

An electronic literature search was conducted using MEDLINE via PubMed (1950 to present), EMBASE (1980 to present), PsycINFO (1985 to present) using OVID SP, and CINAHL (1982 to present) via EBSCO. Initial searches used MEDLINE Major Topic terms [Majr] *Genetic Screening, Mass Screening, Risk Assessment *and screen* which were combined with disease specific terms cancer, diabetes, heart, cardiovasc*, AIDS, HIV, osteoporosis, Huntington*, emotional terms, including emotion*, anxiety, distress*, depression, "quality of life", mood, anger and *"distress thermometer" *including two measures of emotional distress *(GHQ *and *K10) *and publication type *"Randomised Controlled Trial"*. The core search was further limited by excluding the terms *fetal distress, postpartum depression, prenatal, newborn*, and *maternal*, as well as decision aid* and intervention* in the title. Search strategies were tailored to individual databases (available on request).

**Figure 1:**
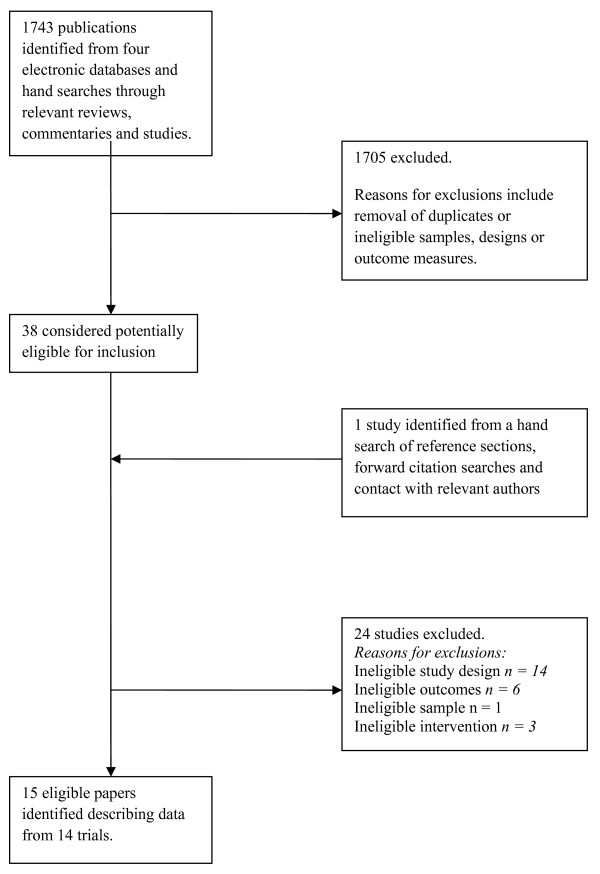
**Flow diagram outlining the search and inclusion stages of the systematic review**.

The search yielded 1743 abstracts which were independently reviewed by two authors, with disagreements resolved by consensus. Thirty-nine papers appearing to meet inclusion criteria based on abstract alone were subject to full-text evaluation, resulting in 15 meeting the eligibility criteria (Figure 1).

Six of the 15 eligible papers contained insufficient data to be included in the meta-analyses and requests for data were made of the authors. Data were provided for three of the six papers; the remaining three were excluded [20-22]. Cross-sectional and cited reference searches were conducted on all papers meeting the inclusion criteria.

### Study selection

Studies considered eligible for the review were randomised controlled trials (RCTs) in which adults in one arm underwent screening or risk assessment and another did not, and in which one measure of emotion was reported on all participants. Emotion was defined broadly to include measures of general mood states as well as emotional well-being.

### Data extraction and quality assessment

Data were extracted (REC) and independently checked (TMM), with disagreements resolved by consensus. Variables of interest included study participants, study design (including number of arms), screening (disease type), emotional outcome (type, time points) and test results (positive or negative).

Risk of bias was assessed by two authors in line with recommended principles [23]. Specific domains examined were: allocation concealment *i.e*. adequate if allocation is concealed from both participants and researchers at least until the point of allocation to groups; randomisation *i.e*. evidence of true randomisation, alternative forms of randomisation, including quasi randomisations, were considered ineligible; baseline comparability *i.e*. comparability of groups at baseline, or alternatively, statistical adjustment for baseline differences; validation of measures *i.e*. evidence of reliability and validity of primary endpoint measures; and follow up *i.e*. outcomes reported on a minimum of 80 percent of participants.

### Data synthesis and analyses

The principal analysis involved comparing randomised groups on emotional outcomes. The secondary analysis involved comparing non-randomised subgroups of those who had undergone screening and received either a positive (elevated risk) or a negative (non elevated risk) test result.

Data were pooled for comparable emotional outcomes with effect sizes presented as standardised mean difference (SMD). The I^2 ^statistic was used to assess the extent of heterogeneity present. Funnel plots were considered, but not reported, due to insufficient meta-analysed study data for meaningful interpretation. Outcomes were categorised as short term (assessed within one month of receipt of test results) and longer term (assessed one month or longer after receipt of test results). Where multiple assessments are reported, the data presented from any one study are those taken as the time point closest to and furthest from receipt of test results.

Sensitivity analyses were conducted to examine the effects of screening approach and disease type. Sensitivity analysis was also conducted to explore the potential impact of attenders versus non-attenders on the primary outcome measures.

Raw data from screened and not-screened groups were combined using a statistical method for one paper [22] to create one overall 'screened' group to facilitate primary analyses.

## Results

### Characteristics of included studies

Of the included 12 papers (Table 1), reporting data from 12 studies, nine involved screening for disease [24-32]and three involved assessing risk of disease [33-35]. For ease of reading, we describe all 12 studies as assessing outcomes of screening. Ten [24-31,33,34] involved randomisation into two groups (screened and not screened), one [35] into three groups (screening plus/minus optional health discussion and not screened) and one [32] into four groups (screened, not screened, risk education and screening plus risk education).

**Table 1:** Characteristics of included studies

Disease and Disease risk	Assessment method	First Author Reference Number	Measures (scale)	Groups (n)	Follow-up	Country
*Screening for presence of Disease*						
**Abdominal Aortic** **Aneurysms (AAA)**	Abdominal Ultrasound	Ashton[24]	State anxiety (STAI^a^), Depression (HADS^b^), QoL (SF-36)	Not-screened (726), Screened (1230), Screen +ve (599), Screen -ve (631)	6 weeks	UK
**Type II diabetes**	Random glucose blood test	Eborall[25]	State anxiety (STAI^a^), Depression (HADS^b^), QoL (SF-36)	Not-screened (444), Screened (2874), Screen +ve (880), Screen -ve (1687)	12-15 months	UK
		Park[30]	State anxiety (STAI^a^), QoL (SF-36)	Not-screened (168), Screened (64)	6 weeks	UK
**Osteoporosis**	Bone mineral density measurement	Torgerson[26]	State anxiety (STAI^a^), QoL (SF-36)	Not-screened (605), Invited (611)	2 years	UK
**Colorectal Cancer**	Faecal occult blood test	Parker[27]	General Distress - Suicide	Not-screened (74,998), Invited(75,253)	14 years	UK
		Whynes[29]	QoL (NHP^d^)	Not-Screened (396), Screened (821)	5 months	UK
**Ovarian Cancer**	CA-125 blood test, transvaginal sonography	Andersen[32]	QoL (SF-36)	Not-Screened (139), Screened (128)	2 years	USA

Disease and Disease risk	Assessment method	First Author Reference Number	Measures (scale)	Groups (n)	Follow-up	Country

*Screening for presence of Disease cont*.						
**Prostate, Lung, Colorectal and Ovarian Cancer Screening**	Digital rectal exam & PSA test (men), CA-125 blood test & transvaginal ultrasound (women), chest x- ray, flexible sigmoidoscopy.	Taylor [31]	QoL (SF-12)	Not-screened (217), Screened (215), Screen +ve (105), Screen -ve (61)	12 months	USA
**Peptic Ulcer**	Helicobacter pylori blood test.	Hansen [28]	QoL (SF-36)	Not-screened (5,612), Screened (4,821)	5 years	Denmark

*Risk Assessment*						
**Lung Cancer**	Genotyping (GSTM1 gene)	Sanderson[33]	Depression, State Anxiety (HADS^b^)	Not-screened (18), Screened (43), Screen +ve (23), Screen -ve (20)	1 week, 2 months	UK
		McBride[34]	Depression (CES-D^e^)	EUC (115), Biomarker Feedback (236)	12 months	USA
**Coronary Heart** **Disease (CHD)**	Additional risk factor for CHD	Christiansen[35]	General Distress (GHQ^c^-12)	Not-screened (396), Screened (904)	5 years	Denmark

^a ^State, Trait, Anxiety Inventory; ^b ^Hospital Anxiety and Depression Scale; ^c ^General Health Questionnaire; ^d ^Nottingham Health Profile; ^e ^Centre for

Epidemiologic Studies Depression Scale.

Four of the 12 studies assessed the impact of screening for cancer [27,29,31,32] with a further two studies assessing the impact of screening for risk of developing lung cancer, based on results of a genetic test [33,34]. Two studies assessed the impact of screening for type II diabetes [25,30]. One study each assessed the impact of screening for abdominal aortic aneurysms [24], osteoporosis [26], peptic ulcer [28], and coronary heart disease [35]. The primary emotional outcomes were depression [24,25,33,34], anxiety [24-26,30,33], and quality of life [24-26,28-32] and general distress [35]. A behavioural index of emotional distress (suicide) was reported in one paper [27].

Anxiety was assessed using the state scale of the state-trait anxiety inventory (STAI) [36] and the anxiety sub-scale of the Hospital Anxiety and Depression Scale (HADS) [37]. Measures assessing depression included the depression sub-scale from the HADS and the Centre for Epidemiological Studies Depression Scale (CES-D) [38]. Quality of life was measured using the Nottingham Health Profile (NHP) [39] and the Short Form Health Survey (SF-12 and SF-36) [40]. General distress was measured using the General Health Questionnaire (GHQ-12) [41].

The 12 studies involved 170,045 participants. The mean ages of participants across the studies ranged from 41 to 69 years. The gender mix of participants ranged from 35% to100% female.

### Quality assessment of included studies

None of the studies included in the review was judged to have a low risk of bias (table available on request). Three of the 12 studies met four of the five criteria [24,28,33] and were judged to have the lowest risk of bias. Five studies met three criteria [24,26,27,30,35], three met two criteria [25,31,34], and one met only one criterion [29]. Validation of measures was the most commonly met criterion and was met by all studies, followed by baseline comparability which was met by 10 of the 12 studies. Allocation concealment of participants to trial arm prior to randomisation was the most common risk of bias, with only three studies meeting this criterion.

Of the 12 included studies, nine involved screening for disease [24-32] and three involved assessing risk of disease [33-35]. For ease of reading, we describe all 12 studies as assessing outcomes of screening (Table 2).

**Table 2:** Analysis of emotional impact of screening

Measure	Time	Comparison	k	n	Z	*p*	SMD	95%CI	I^2^
Anxiety	< 4 weeks	Screened *vs*. Not-Screened	1	61	1.76	0.08	-0.50	-1.06 to 0.06	-
	> 4 weeks	Screened *vs*. Not-Screened	5	5190	0.10	0.92	0.01	-0.10 to 0.11	58%
	< 4 weeks	Screen +ve *vs*. Screen -ve	2	2511	1.44	0.15	0.06	-0.02 to 0.14	0%
	> 4 weeks	Screen +ve *vs*. Screen -ve	2	1273	0.20	0.84	-0.05	-0.53 to 0.44	64%

Depression	< 4 weeks	Screened *vs*. Not-Screened	1	61	1.74	0.08	-0.50	-1.05 to 0.06	-
	> 4 weeks	Screened *vs*. Not-Screened	4	4342	0.46	0.65	0.04	-0.12 to 0.20	73%
	< 4 weeks	Screen +ve *vs*. Screen -ve	2	3204	1.86	0.06	0.07	0.00 to 0.14	0%
	> 4 weeks	Screen +ve *vs*. Screen -ve	2	1273	1.37	0.17	0.08	-0.03 to 0.19	0%

QoL Mental	> 4 weeks	Screened *vs*. Not-Screened	5	14,199	0.57	0.57	0.01	-0.02 to 0.04	88%
	> 4 weeks	Screen +ve *vs*. Screen -ve	2	1379	0.24	0.81	0.06	-0.45 to 0.57	0%

QoL Self-Assessed Health	> 4 weeks	Screened *vs*. Not-Screened	4	15,199	0.18	0.85	0.00	-0.04 to 0.03	0%

Note. CI = Confidence Interval; SMD = Standardised Mean Difference; I^2 ^= homogeneity test; k = number of studies contributing to meta-analyses.

Data were pooled for studies reporting the same emotional outcome. Pooling was conducted separately for emotional outcomes assessed in the short term (less than one month after screening) and those assessed in the longer term (beyond one month). The primary comparison was between randomised groups, namely those undergoing screening and those not undergoing screening. Secondary analysis involved non- randomised comparisons between those who had undergone screening and received a positive test result (*i.e*. one indicating an elevated risk of disease) and those who had undergone screening and received a negative test result (*i.e*. one indicating no elevated risk of disease).

#### Anxiety

Only one study assessed the impact of screening on anxiety within four weeks of screening (n = 61) [33]. The pooled SMD was -0.50 (95% CI -1.06, 0.06) indicating no short term impact of screening on anxiety. Five studies [24-26,30,33] assessed longer term anxiety (n = 5,910). The pooled SMD was 0.01 (95% CI -0.10, 0.11), indicating no adverse effect of screening upon anxiety beyond four weeks of screening.

Two studies [25,33] provided sufficient data to enable quantitative synthesis of short term anxiety by screening test outcome (n = 2,511). The pooled SMD was 0.06 (95% CI -0.02, 0.14) indicating no evidence of short term anxiety in those receiving positive test results compared with those receiving negative test results.

Two studies [24,33] provided sufficient data to enable quantitative synthesis of longer term anxiety by screening test outcome (n = 1,273). The pooled estimate of the overall standardised difference was -0.05 (95% CI -0.53, 0.44) indicating no evidence of longer term anxiety for those receiving positive test results compared with those receiving negative results.

#### Depression

Only one study assessed the impact of screening on depression within four weeks of screening (n = 61) [33]. The SMD was -0.50 (95% CI -1.05, 0.06), indicating no short term impact of screening on depression. Four studies [24,25,33,34] assessed depression four weeks or longer after screening (n = 4,342). The pooled SMD was 0.04 (95% CI -0.12, 0.20), indicating no adverse effect of screening upon depression beyond four weeks of screening.

Two studies [25,33] provided sufficient data to enable quantitative synthesis of short term depression by screening test outcome (n = 3,204). The pooled estimate of the overall standardised difference was 0.07 (95% CI 0.00, 0.14), providing marginal evidence for raised short term depression in those screening positive. Two studies [24,33] provided sufficient data to enable quantitative synthesis of longer term depression by screening test outcome (n = 1,273). The pooled estimate of the overall standardised difference was 0.08 (95% CI -0.03, 0.19), indicating no evidence of increased depression, one month or more after screening.

#### Quality of Life

There were insufficient data to examine the impact of screening on quality of life within four weeks of screening.

Five studies [24,26,28,31,32] assessed mental quality of life four weeks or longer after screening (n = 14,199). The pooled SMD was 0.01 (95% CI -0.02, 0.04) indicating no adverse effect of screening upon mental quality of life beyond four weeks of screening. Four studies [25,26,28,30] examined the self-assessed health subscales of quality of life four weeks or longer after screening (n = 15,119). The pooled SMD was 0.00 (95% CI -0.04, 0.03) indicating no adverse effect of screening upon general health beyond four weeks of screening. One study reported quality of life using the Nottingham Health Profile (n = 415) [29]. No adverse effect of screening was found with standardised mean differences on scales of energy -0.01 (-0.21, 0.18), emotional reactions 0.04 (-0.15, 0.23), pain -0.08 (-0.28, 0.11), physical mobility 0.02 (-0.17, 0.22) sleep 0.05 (-0.14, 0.24) or social isolation 0.11 (-0.08, 0.31) respectively.

No data were available to examine quality of life in line with test outcome at less than four weeks after receipt of test results. Two studies [24,31] provided sufficient data to enable quantitative synthesis of mental quality of life by screening test outcome in the longer term (n = 1,379). The pooled estimate of the overall standardised difference was 0.06 (95% CI -0.45, 0.57) indicating little impact of test result on quality of life (mental).

#### General Distress

No data were available to examine the impact of screening on general distress at less than four weeks after receipt of test results. Only one study assessed general distress in the longer term (n = 784) [35]. The SMD was -0.03 (95% CI -0.15, 0.09), indicating no short term impact of screening on general distress. One study assessed suicide, a behavioural index of emotional distress [27], with no reported differences found between study arms (OR 0.91 95% CI 0.61, 1.34).

### Sensitivity analysis

Sensitivity analysis was conducted to explore the potential impact of screening approach (screening for disease versus estimating disease risk) on the overall results. Removal of studies estimating disease risk [33-35] had no impact on the primary outcome. Sensitivity analysis was also conducted to assess the impact of measurements on the primary outcome measure as assessed in both non-attenders and attenders versus attenders only. Removal of studies assessing the both non-attenders and attenders [26] had no impact on the primary outcome. Sensitivity analysis was also conducted to investigate the impact of disease type on overall results *(i.e*. those screening for cancer versus other diseases). Removal of these studies had no impact on the primary outcome with the exception of mental quality of life between screen positive and screen negative groups.

## Discussion

We found no evidence that undergoing screening has an adverse emotional impact when assessed four or more weeks after screening. Too few studies assessed outcomes before four weeks to comment on the shorter term emotional impact of screening. Subgroup comparisons between those receiving positive and those receiving negative test results reveal a small, transient impact of being informed of an elevated risk of disease, discernible on depression.

These findings are consistent with psychological theories of self-regulation which describe the complex ways in which humans maintain emotional equilibrium while managing threats [42]. Managing threats in the current context includes engaging in behaviours to reduce threats to health, as well as using cognitive strategies to minimise the severity or likelihood of the threat. Both of these can reduce emotional distress. Emotional distress is a common and adaptive initial response to risk notification, but, as replicated in the current review, this has usually dissipated by one month [3]. These findings also reflect those from existing, but disparate and methodologically less robust reviews, which have focused on screening for inherited or genetic conditions [14-16,43,44]. Only one previous review has assessed the impact of screening for a broader range of conditions [3].

The strength of our review is that it is the first systematic review with meta- analysis involving comparisons of emotional outcomes assessed in RCTs. The findings are therefore more robust than those from existing reviews. The findings of the review may, however, reflect some bias evident in the conduct of the reviewed studies. This includes bias from sampling as well as from the measures used. Only half of the studies reported outcomes on 80% or more of participants. Consequently, pooled comparisons often contain a small number of studies. Examination of follow-up between randomised arms, however, revealed similar levels of attrition between screened and not-screened groups. Of concern is that those lost to follow-up may have been those who experienced higher levels of distress than those who remained in the studies, for which there is some evidence [12,45]. The studies also varied in the populations sampled in the intervention arm. In some trials, this included attenders and non-attenders [26,27] and in others, only attenders [21,22,25-30,33,34]. The measures used to assess emotional outcomes may have been inappropriate or insufficiently sensitive to detect adverse emotional outcomes. All the included studies used standardised measures of generalised emotional functioning. Such measures are not designed to detect subtle changes, such as worry about health, that do not affect general mood states. The claims that can be made from the current review therefore concern general levels of functioning and not more subtle impacts of screening on worries specifically related to health.

**Figure 2:**
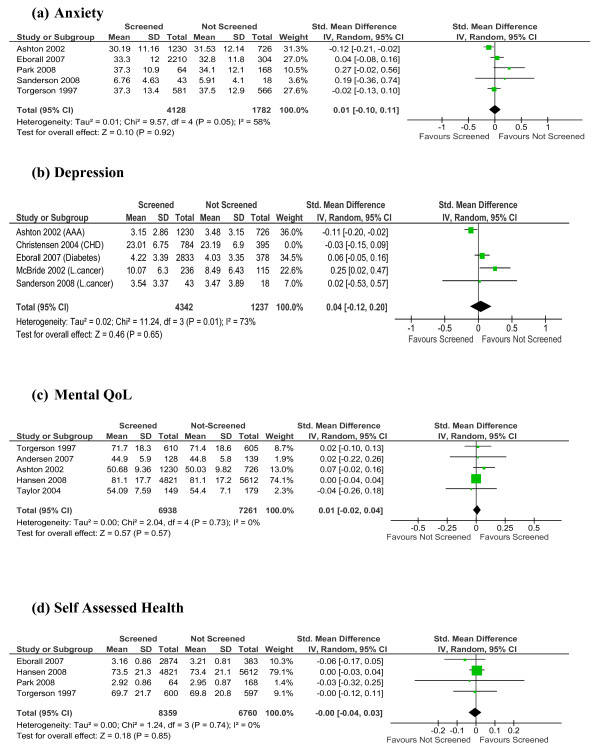
**Forest plots depicting the impact after four weeks of screening**.

The review was also limited by the relatively large heterogeneity of the included studies, as reflected in the I^2 ^scores (Figure 2), particularly those above 50%. The studies varied greatly in the demographic characteristics of participants, the diseases for which screening was being undertaken as well as the processes of screening. Pooled comparisons combined studies screening for disparate disease types which may have impacted on the findings. Sensitivity analysis revealed no differences in overall results by disease type with the exception of mental quality of life between screened versus non screened groups. Future reviews might consider the impact of disease specific factors such as prevalence and severity, and test specific factors such as sensitivity and specificity factors on emotional responses. One of the studies included was tailored specifically for smokers [33], a further two involved multiple screening procedures [31,32], one of which involved screening for the risk of several diseases [31]. The studies are likely to have varied in the information provided and support offered to participants, but insufficient detail was provided to allow subgroup analysis on this in the current review. Existing evidence suggests this is likely to have affected emotional responses, particularly in the short-term [46-50].

## Conclusion

The results of this review reduce uncertainty about emotional outcomes and suggest that, provided other criteria for screening are met [51], there are few if any grounds for not screening on the basis that it has adverse longer term emotional outcomes.

## PubMed Search Strategy

("Genetic Screening"[Majr] OR "Mass Screening"[Majr] OR "Risk Assessment"[Majr] OR ((cancer OR diabetes OR heart OR cardiac OR cardioavasc* OR AIDS OR HIV OR osteoporosis OR Huntington*) AND screen*)) AND (emotion* OR anxiety OR distress* OR depression OR mood or anger or GHQ OR K10 OR (quality of life)) NOT (fetal distress OR postpartum depression OR prenatal OR newborn OR maternal) NOT decision aid*[ti] NOT intervention*[ti] AND ((Randomized Controlled Trial[ptyp]).

## Competing interests

All authors have completed the Unified Competing Interest form and declare that all authors (REC, LML & TMM) have no financial interests that may be relevant to the submitted work.

## Authors' contributions

TMM has had full access to all of the data in the study and takes responsibility for the integrity of the data and the accuracy of the data analysis. Writing the protocol: TMM, REC. Developing the search strategy: LML & TMM. Searching for trials: LML, REC. Selecting trials: TMM, REC. Data entry: REC. Analysis: REC. Interpreting analysis: TMM, REC, LML. Drafting final review: All.

## Pre-publication history

The pre-publication history for this paper can be accessed here:

http://www.biomedcentral.com/1471-2458/11/603/prepub

## References

[B1] Holland WW Stewart S Screening in health care: benefit or bane? 1990 Nuffield Provincial Hospitals Trust

[B2] Haynes RB Sackett DL Taylor DW Gibson ES Johnson AL Increased Absenteeism from Work after Detection and Labeling of Hypertensive Patients New England Journal of Medicine 1978 299 14 741 744 10.1056/NEJM197810052991403 692548

[B3] Shaw C Abrams K Marteau TM Psychological impact of predicting individuals' risks of illness: a systematic review Social Science & Medicine 1999 49 12 1571 1598 10.1016/S0277-9536(99)00244-0 10574231

[B4] Michie S Marteau TM Predictive genetic testing in children: the need for psychological research British Journal of Health Psychology 1996 1 3 14

[B5] Wardle J Taylor T Sutton S Atkin W Does publicity about cancer screening raise fear of cancer? Randomised trial of the psychological effect of information about cancer screening British Medical Journal 1999 319 7216 1037 1038 10521195 10.1136/bmj.319.7216.1037 PMC32262

[B6] Fallowfield LJ Rodway A Baum M What Are the Psychological-Factors Influencing Attendance, Nonattendance and Re-Attendance at a Breast Screening Center Journal of the Royal Society of Medicine 1990 83 9 547 551 2213798 10.1177/014107689008300905 PMC1292810

[B7] Cockburn J Staples M Hurley SF Deluise T Psychological consequences of screening mammography Journal of Med Screen 1994 1 7 12 10.1177/096914139400100104 8790480

[B8] Tercyak KP Lerman C Peshkin BN Hughes C Main D Isaacs C Schwartz MD Effects of coping style and BRCA1 and BRCA2 test results on anxiety among women participating in genetic counseling and testing for breast and ovarian cancer risk Health Psychology 2001 20 3 217 222 11403219

[B9] Yerkes RM Dodson JD The relation of strength of stimulus to rapidity of habit-formation J Comp Neurol Psycho 1908 18 5 459 482 10.1002/cne.920180503

[B10] Eich E Kihlstrom JF Bower GH Forgas JP Niedenthal PM Cognition and Emotion: Counterpoints: Cognition, Memory, and Language 2000 Oxford: Oxford University Press

[B11] Kash KM Holland JC Halper MS Miller DG Psychological distress and surveillance behaviors of women with a family history of breast cancer J Natl Cancer Inst 1992 84 1 24 30 10.1093/jnci/84.1.24 1738170

[B12] Lerman C Schwartz M Adherence and psychological adjustment among women at high risk for breast cancer Breast Cancer Res Treat 1993 28 2 145 155 10.1007/BF00666427 8173067

[B13] French DP Maissi E Marteau TM The psychological costs of inadequate cervical smear test results: Three-month follow-up Psycho-Oncology 2006 15 6 498 508 10.1002/pon.980 16184520

[B14] Heshka JT Palleschi C Howley H Wilson B Wells PS A systematic review of perceived risks, psychological and behavioral impacts of genetic testing Genetics in Medicine 2008 10 19 32 10.1097/GIM.0b013e31815f524f 18197053

[B15] Butow PN Lobb EA Meiser B Barratt A Tucker KM Psychological outcomes and risk perception after genetic testing and counselling in breast cancer: a systematic review Medical Journal of Australia 2003 178 2 77 81 12526728 10.5694/j.1326-5377.2003.tb05069.x

[B16] Broadstock M Michie S Marteau T Psychological consequences of predictive genetic testing: a systematic review European Journal of Human Genetics 2000 8 10 731 738 10.1038/sj.ejhg.5200532 11039571

[B17] Rogstad KE The psychological impact of abnormal cytology and colposcopy BJOG: an International Journal of Obstetrics and Gynaecology 2002 109 4 364 368 10.1111/j.1471-0528.2002.99023.x 12013155

[B18] Johnson JN Should we screen for aortic aneurysm? No BMJ 2008 336 7649 863 10.1136/bmj.39514.494167.AD 18420692 PMC2323076

[B19] Kane RA Kane RL Effect of Genetic Testing for Risk of Alzheimer's Disease New England Journal of Medicine 2009 361 3 298 299 10.1056/NEJMe0903449 19605835

[B20] Lerman C Gold K Audrain J Lin T Boyd N Orleans C Wilfond B Louben G Caporaso N Incorporating biomarkers of exposure and genetic susceptibility into smoking cessation treatment: Effects on smoking-related cognitions, emotions, and behavior change Health Psychology 1997 16 87 99 9028818 10.1037//0278-6133.16.1.87

[B21] Audrain J Boyd NR Roth J Main D Caporaso NF Lerman C Genetic susceptibility testing in smoking-cessation treatment: one-year outcomes of a randomized trial Addictive Behaviors 1997 741 751 10.1016/s0306-4603(97)00060-9 9426791

[B22] Lindholt JS Vammen S Fasting H Henneberg EW Psychological consequences of screening for abdominal aortic aneurysm and conservative treatment of small abdominal aortic aneurysms Eur J Vasc Endovasc 2000 20 1 79 83 10.1053/ejvs.1999.1087 10906303

[B23] Higgins J Green SP Cochrane handbook for systematic reviews of interventions. sept 2008 edition 2008 Oxford: Wiley-Blackwell

[B24] Ashton HA Buxton MJ Day NE Kim LG Marteau TM Scott RAP Thomspon SG Walker NM Multicentre Aneurysm Screening S The Multicentre Aneurysm Screening Study (MASS) into the effect of abdominal aortic aneurysm screening on mortality in men: a randomised controlled trial Lancet 2002 360 9345 1531 1539 10.1016/S0140-6736(02)11522-4 12443589

[B25] Eborall HC Griffin SJ Prevost AT Kinmonth AL French DP Sutton S Psychological impact of screening for type 2 diabetes: controlled trial and comparative study embedded in the ADDITION (Cambridge) randomised controlled trial BMJ: British Medical Journal 2007 335 7618 486 493 10.1136/bmj.39303.723449.55 17761995 PMC1971192

[B26] Torgerson DJ Thomas RE Campbell MK Reid DM Randomized trial of osteoporosis screening. Use of hormone replacement therapy and quality-of-life results Archives of internal medicine 1997 2121 2125 10.1001/archinte.157.18.2121 9382669

[B27] Parker MA Robinson MH Scholefield JH Hardcastle JD Psychiatric morbidity and screening for colorectal cancer Journal of medical screening 2002 7 10 10.1136/jms.9.1.7 11943790

[B28] Hansen JM Wildner-Christensen M Hallas J Schaffalitzky de Muckadell OB Effect of a community screening for Helicobacter pylori: a 5-Yr follow-up study The American journal of gastroenterology 2008 103 1106 1113 10.1111/j.1572-0241.2007.01770.x 18445098

[B29] Whynes DK Neilson AR Robinson MHE Hardcastle JD Colorectal cancer screening and quality of life Quality of Life Research 1994 3 3 191 198 10.1007/BF00435384 7920493

[B30] Park P Simmons RK Prevost AT Griffin SJ Screening for type 2 diabetes is feasible, acceptable, but associated with increased short-term anxiety: A randomised controlled trial in British general practice BMC Public Health 2008 8 350 359 10.1186/1471-2458-8-350 18840266 PMC2567326

[B31] Taylor KL Shelby R Gelmann E McGuire C Quality of life and trial adherence among participants in the prostate, lung, colorectal, and ovarian cancer screening trial Journal of the National Cancer Institute 2004 96 14 1083 1094 10.1093/jnci/djh194 15265970

[B32] Andersen MR Drescher CW Zheng Y Bowen DJ Wilson S Young A McIntosh M Mahony BS Lowe KA Urban N Changes in cancer worry associated with participation in ovarian cancer screening Psychooncology 2007 16 9 814 820 10.1002/pon.1151 17225260

[B33] Sanderson SC Humphries SE Hubbart C Hughes E Jarvis MJ Wardle J Psychological and behavioural impact of genetic testing smokers for lung cancer risk - A phase II exploratory trial Journal of Health Psychology 2008 13 4 481 494 10.1177/1359105308088519 18420756

[B34] McBride CM Bepler G Lipkus IM Lyna P Samsa G Albright J Datta S Rimer BK Incorporating genetic susceptibility feedback into a smoking cessation program for African-American smokers with low income Cancer Epidemiology Biomarkers & Prevention 2002 11 6 521 528 12050092

[B35] Christensen B Engberg M Lauritzen T No long-term psychological reaction to information about increased risk of coronary heart disease in general practice. [Article] European Journal of Cardiovascular Prevention & Rehabilitation: official journal of the European Society of Cardiology, Working Groups on Epidemiology & Prevention and Cardiac Rehabilitation and Exercise Physiology 2004 11 3 239 243 15179107 10.1097/01.hjr.0000129739.30593.23

[B36] Spielberger CD Gorsuch RL Lushene RE STAI: Manual for the State-Trait Anxiety Inventory (Self - Evaluation Questionnaire) 1970 Palo Alto, CA Consulting Psychologists

[B37] Zigmond AS Snaith RP The Hospital Anxiety and Depression Scale Acta Psychiat Scand 1983 67 6 361 370 10.1111/j.1600-0447.1983.tb09716.x 6880820

[B38] Radloff LS The CES-D Scale: A self report depression scale for research in the general population Applied Psychological Measurement 1977 1 3 385 401 10.1177/014662167700100306

[B39] Hunt SM Mckenna SP Mcewen J Williams J Papp E The Nottingham Health Profile - Subjective Health-Status and Medical Consultations Soc Sci Med-Med Soc 1981 15 3 221 229 10.1016/0271-7123(81)90005-5 6973203

[B40] Ware JE Sherbourne CD The Mos 36-Item Short-Form Health Survey (Sf-36) .1. Conceptual-Framework and Item Selection Medical Care 1992 30 6 473 483 10.1097/00005650-199206000-00002 1593914

[B41] Goldberg DP Manual of the General Health Questionnaire 1978 Windsor, England: NFER Publishing

[B42] Carver C Scheier M Baumeister R Control Theory: A useful conceptual framework for personality-social, clinical and health psychology The Self in Social Psychology 1999 Philadelphia: Psychology Press 299 316 7134324

[B43] Meiser B Psychological impact of genetic testing for cancer susceptibility: An update of the literature Psycho-Oncology 2005 14 12 1060 1074 10.1002/pon.933 15937976

[B44] Brett J Bankhead C Henderson B Watson E Austoker J The psychological impact of mammographic screening. A systematic review Psycho-Oncology 2005 14 11 917 938 10.1002/pon.904 15786514

[B45] Timman R Roos R Maat-Kievit A Tibben A Adverse effects of predictive testing for Huntington disease underestimated: Long-term effects 7-10 years after the test Health Psychology 2004 23 2 189 197 15008664 10.1037/0278-6133.23.2.189

[B46] Stewart DE Lickrish GM Sierra S Parkin H The Effect of Educational Brochures on Knowledge and Emotional Distress in Women with Abnormal Papanicolaou Smears Obstetrics And Gynecology 1993 81 2 280 282 8423964

[B47] Rudd P Price MG Graham LE Beilstein BA Tarbell SJ Bacchetti P Fortmann SP Consequences of worksite hypertension screening. Changes in absenteeism Hypertension 1987 10 4 425 436 3653971 10.1161/01.hyp.10.4.425

[B48] Antoni MH Lehman JM Kilbourn KM Boyers AE Culver JL Alferi SM Yount SE McGregor BA Arena PL Harris SD Cognitive-behavioral stress management intervention decreases the prevalence of depression and enhances benefit finding among women under treatment for early-stage breast cancer Health Psychol 2001 20 1 20 32 11199062 10.1037//0278-6133.20.1.20

[B49] Perry S Fishman B Jacobsberg L Young J Frances A Effectiveness of Psychoeducational Interventions in Reducing Emotional Distress after Human-Immunodeficiency-Virus Antibody Testing Archives of General Psychiatry 1991 48 2 143 147 1989570 10.1001/archpsyc.1991.01810260051008

[B50] Wilkinson C Jones J McBride J Anxiety caused by abnormal result of cervical smear test: A controlled trial British Medical Journal 1990 300 6722 440 440 10.1136/bmj.300.6722.440 2107898 PMC1662255

[B51] Wilson JM Principles of screening for disease Proc R Soc Med 1971 64 12 1255 1256 5131278 10.1177/003591577106401238 PMC1813182

