# Correction: Emotional impact of screening: A systematic review and meta-analysis

**DOI:** 10.1186/1471-2458-11-752

**Published:** 2011-09-30

**Authors:** Ruth E Collins, Laureen M Lopez, Theresa M Marteau

**Affiliations:** 1 Kings College London, Department of Psychology, Health Psychology Section, 5th Floor Bermondsey Wing, Guy's Campus, London, SE1 9RT, UK; 2 Clinical Sciences FHI, PO Box 13950, Research Triangle Park, NC 27709, USA

## Correction

After the publication of this work [1], we became aware that two of the twelve studies included in the review [refs 25 and 30] were erroneously described as not having presented outcomes by randomisation (*ie *those randomised to screening *vs *those randomised not to be screened). Instead they were described as having presented outcomes according to those who were randomised and who attended *vs *those randomised not to be screened).

We have now extracted the correct data from these two studies which resulted in revised pooled Standardised Mean Differences (SMDs). None of the conclusions reached in the uncorrected version of the paper has been revised in the light of these changes.

We describe here the changes arising from this error as shown in corrected form in the accompanying revised Tables 1 and 2 and Figure 2.

(i) The number of participants included has now been amended to reflect the altered data extracted from studies 25 and 30, altering this from 170,045 to 170,277 participants, as shown in Table 1 and Table 2.

(ii) The SMDs from the meta-analyses have been recalculated resulting in changes to the pooled SMDs now shown in Figure 2 where corrections have been made to the data for sections (a) (b) and (d).

(iii) The sensitivity analyses have been redone so that studies 25 and 30, in addition to study 26, were removed to assess the impact of measurements in the primary outcome being made in all those randomised to be screened regardless of whether or not they attended (as opposed to those randomised and who attended).

The inclusion of the two further studies did not alter the outcome of this sensitivity analysis, which reveals no impact on the primary outcome.

In making these changes we became aware of three other errors that had not been corrected at final draft stage of the publishing process. These are described below:

(iv) In the abstract, we erroneously reported the number of studies assessing depression as four. It should be five.

(v) Data from [25] were erroneously included in pooled estimates of short term anxiety and depression by screening test outcome.

(vi) The references listed as including data from attenders and non attenders in the intervention arm should now read: [25,26,27 & 30] and the reference to those including only attenders should correctly read: [24,28,29,31-35].

We regret any inconvenience that these corrections might have caused. We wish to thank Dr. Simon Griffin for bringing this matter to our attention in relation to reference 30, resulting in re-checking of all data extracted from the included studies.

## Pre-publication history

The pre-publication history for this paper can be accessed here:

http://www.biomedcentral.com/1471-2458/11/752/prepub

## Supplementary Material

Additional file 1 **Revised tables**. Click here for file

Additional file 2 Revised Figure 2. Click here for file
