# Supplementary material for: Correction: Emotional impact of screening: A systematic review and meta-analysis
Source: BMC Public Health. 2011 Sep 30;11:752. doi: 10.1186/1471-2458-11-752 (PMC3201924; doi:10.1186/1471-2458-11-752)
Supplement: Additional file 1 — Revised tables. [file 1471-2458-11-752-S1.DOC]

| **Table 1: Characteristics of included studies** | | | | | | |  |
| --- | --- | --- | --- | --- | --- | --- | --- |
| Disease and Disease risk | Assessment method | First Author  Reference Number | Measures  (scale) | Groups (n) | Follow-up | Country | |
| *Screening for presence of Disease* | | |  |  |  |  | |
| **Abdominal Aortic**  **Aneurysms (AAA)** | Abdominal  Ultrasound | Ashton^24^ | State anxiety (STAIa),  Depression (HADSb),  QoL (SF-36) | Not-screened (726), Screened (1230), Screen +ve (599), Screen –ve (631) | 6 weeks | UK | |
| **Type II diabetes** | Random glucose blood test | Eborall^25^ | State anxiety (STAIa), Depression (HADSb), QoL (SF-36) | Not-invited (444), Invited (3093), Screen +ve (880), Screen –ve (1687) | 12-15  months | UK | |
|  |  | Park^30^ | State anxiety (STAIa), QoL (SF-36) | Not-invited (168), Invited (77) | 6 weeks | UK | |
| **Osteoporosis** | Bone mineral density measurement | Torgerson^26^ | State anxiety (STAIa), QoL (SF-36) | Not-screened (605), Invited (611) | 2 years | UK | |
| **Colorectal Cancer** | Faecal occult  blood test | Parker^27^ | General Distress - Suicide | Not-screened (74,998), Invited(75,253) | 14 years | UK | |
|  |  | Whynes^29^ | QoL (NHPd) | Not-Screened (396), Screened (821) | 5 months | UK | |
| **Ovarian Cancer** | CA-125 blood test, transvaginal sonography | Andersen^32^ | QoL (SF-36) | Not-Screened (139), Screened (128) | 2 years | USA | |

| **Prostate, Lung, Colorectal and Ovarian Cancer Screening** | Digital rectal exam & PSA test (men), CA-125 blood test & transvaginal ultrasound (women), chest x- ray, flexible sigmoidoscopy. | Taylor 31 | QoL (SF-12) | Not-screened (217), Screened (215), Screen +ve (105), Screen -ve (61) | 12 months | USA | |
| --- | --- | --- | --- | --- | --- | --- | --- |
| **Peptic Ulcer** | Helicobacter  pylori blood test. | Hansen 28 | QoL (SF-36) | Not-screened (5,612),  Screened (4,821) | 5 years | Denmark | |
| *Risk Assessment* | | | | | | | |
| **Lung Cancer** | Genotyping  (GSTM1 gene) | Sanderson^33^ | Depression, State Anxiety (HADSb) | Not-screened (18), Screened (43), Screen +ve (23),  Screen –ve (20) | 1 week, 2  months | UK | |
|  |  | McBride^34^ | Depression  (CES-De) | EUC (115), Biomarker Feedback (236) | 12 months | USA | |
| **Coronary Heart**  **Disease (CHD)** | Additional risk factor for CHD | Christensen^35^ | General Distress (GHQc-12) | Not-screened (396), Screened (904) | 5 years | Denmark | |
| a State, Trait, Anxiety Inventory; b Hospital Anxiety and Depression Scale; c General Health Questionnaire; d Nottingham Health Profile; e Centre for  Epidemiologic Studies Depression Scale. | | | | | | |  |

| **Table 2: Analysis of emotional impact of screening** | | | | | | | | | |
| --- | --- | --- | --- | --- | --- | --- | --- | --- | --- |
| **Measure** | **Time** | **Comparison** | **k** | **n** | **Z** | ***p*** | **SMD** | **95%CI** | **I^2^** |
| Anxiety | <4 weeks | Screened *vs.* Not-Screened | 1 | 61 | 1.76 | 0.08 | -0.50 | -1.06 to 0.06 | - |
|  | >4 weeks | Screened *vs.* Not-Screened | 5 | 6090 | 0.02 | 0.98 | -0.00 | -0.11 to 0.11 | 58% |
|  | <4 weeks | Screen +ve *vs.* Screen -ve | 2 | 2511 | 1.44 | 0.15 | 0.06 | -0.02 to 0.14 | 0% |
|  | >4weeks | Screen +ve *vs.* Screen –ve | 2 | 1273 | 0.20 | 0.84 | -0.05 | -0.53 to 0.44 | 64% |
| Depression | <4 weeks | Screened *vs.* Not-Screened | 1 | 61 | 1.74 | 0.08 | -0.50 | -1.05 to 0.06 | - |
|  | >4 weeks | Screened *vs.* Not-Screened | 5 | 6974 | 0.34 | 0.74 | 0.02 | -0.10 to 0.14 | 67% |
|  | <4 weeks | Screen +ve *vs.* Screen -ve | 2 | 3204 | 1.86 | 0.06 | 0.07 | 0.00 to 0.14 | 0% |
|  | >4weeks | Screen +ve *vs.* Screen –ve | 2 | 1273 | 1.37 | 0.17 | 0.08 | -0.03 to 0.19 | 0% |
| QoL Mental | >4 weeks | Screened *vs.* Not-Screened | 5 | 14,199 | 0.57 | 0.57 | 0.01 | -0.02 to 0.04 | 88% |
|  | >4weeks | Screen +ve *vs.* Screen –ve | 2 | 1379 | 0.24 | 0.81 | 0.06 | -0.45 to 0.57 | 0% |
| QoL Self-Assessed Health | >4weeks | Screened *vs.* Not-Screened | 4 | 15,351 | 0.20 | 0.84 | -0.00 | -0.04 to 0.03 | 0% |
| Note. CI = Confidence Interval; SMD = Standardised Mean Difference; I^2^ = homogeneity test; k = number of studies contributing to meta-analyses. | | | | | | | | | |
