# Supplementary material for: Correction: Emotional impact of screening: A systematic review and meta-analysis
Source: BMC Public Health. 2011 Sep 30;11:752. doi: 10.1186/1471-2458-11-752 (PMC3201924; doi:10.1186/1471-2458-11-752)
Supplement: Additional file 2 — Revised Figure 2. [file 1471-2458-11-752-S2.DOC]

(a) Anxiety(b) Depression

(c) Mental Quality of Life

(d) Self Assessed Health

Study or Subgroup

Eborall 2007 (Diabetes**)**

Hansen 2008 (P.Ulcer)

Park 2008 (Diabetes)

Torgerson 1997(Osteop)

Total (95% CI)

Heterogeneity: Tau² = 0.00; Chi² = 1.70, df = 3 (P = 0.64); I² = 0%

Test for overall effect: Z = 0.20 (P = 0.84)

Mean

3.15

73.5

2.97

69.7

SD

0.87

21.3

0.86

21.7

Total

3093

4821

77

600

8591

Mean

3.21

73.4

2.95

69.8

SD

0.81

21.1

0.87

20.8

Total

383

5612

168

597

6760

**Weight**

10.4%

78.9%

1.6%

9.1%

100.0%

IV, Random, 95% CI

**-**0.07 [-0.18, 0.04]

0.00 [-0.03, 0.04]

0.02 [-0.25, 0.29]

**-**0.00 [-0.12, 0.11]

-0.00 [-0.04, 0.03]

Screened

Not Screened

Std. Mean Difference

Std. Mean Difference

IV, Random, 95% CI

**-**0.2

**-**0.1

0

0.1

0.2

Favours Not Screened

Favours Screened
